# Considering lead-time bias in evaluating the effectiveness of lung cancer screening with real-world data

**DOI:** 10.1038/s41598-021-91852-6

**Published:** 2021-06-09

**Authors:** Szu-Chun Yang, Jung-Der Wang, Shi-Yi Wang

**Affiliations:** 1 Department of Internal Medicine, National Cheng Kung University Hospital, College of Medicine, National Cheng Kung University, Tainan, Taiwan; 2 Department of Chronic Disease Epidemiology, Yale University School of Public Health, New Haven, CT USA; 3 Department of Public Health, College of Medicine, National Cheng Kung University, 1 University Road, 701 Tainan, Taiwan; 4 Cancer Outcomes, Public Policy, and Effectiveness Research Center, Yale University School of Medicine, New Haven, CT USA

**Keywords:** Outcomes research, Health policy, Population screening, Health services, Health care economics

## Abstract

Low-dose computed tomography screening can be used to diagnose lung cancer at a younger age compared to no screening. Real-world studies observing mortality after lung cancer diagnosis are subject to lead-time bias. This study developed a method using a nationwide cancer registry and stage shift from trial for the adjustment of lead-time bias. 78,897 Taiwanese nationwide lung cancer patients aged 55–82 were matched with 788,820 referents randomly selected from the general population at a ratio of 1:10 by age, sex, calendar year, and comorbidities, to estimate the pathology- and stage-specific life expectancy (LE). Loss-of-LE is the difference between the LE of cancer patients and that of referents. By multiplying LE and loss-of-LE by the pathology and stage shift in the National Lung Screening Trial (NLST), we compared the effectiveness of cancer screening measured by LE gained and loss-of-LE saved. The mean LEs of stage IA and IV adenocarcinoma were 14.5 and 1.9 years, respectively, indicating a LE gain of 12.6 years. However, the mean loss-of-LEs of stage IA and IV adenocarcinoma were 3.7 and 15.1 years, respectively, with a saving of only 11.4 years, implying an adjustment of different distributions of age, sex, and calendar year of diagnosis from stage shift and a reduction in lead-time bias. Applying such estimations on the results of 10,000 participants with the same pathology and stage shift in the NLST, the benefit of screening using LE gained would be 410.3 (95% prediction interval: 328.4 to 503.3) years. It became 297.1 (95% prediction interval: 187.8 to 396.4) years when using loss-of-LE saved, indicating the former approach would overestimate the effectiveness by 38%. Our approach of multiplying loss-of-LE by pathology and stage shift to estimate loss-of-LE saved could adjust for different distributions of age, sex, and calendar year at early diagnosis and reduce lead-time bias.

## Introduction

Low-dose computed tomography (LDCT) screening has been proven as an effective way to reduce lung-cancer mortality^1,2^. The U.S. Preventive Services Task Force began recommending annual LDCT in 2014^3^, and the Centers for Medicare & Medicaid Services began reimbursing the procedure from 2015^4^. However, it remains a challenge to evaluate its effectiveness in real-world practice. Specifically, lead time is defined as the period between the time of detection by screening and the time at which the disease would have been diagnosed in the absence of screening^5^. Lead-time bias denotes that screening makes the diagnoses earlier but does not delay the date of death^6^. Although clinical trials have started observing mortality from the date of randomization to eliminate differential lead time, real-world studies inspecting the outcome after lung cancer diagnosis are subject to lead-time bias. Lead-time bias must be adjusted to avoid overestimation of effectiveness.

Researchers have attempted to quantify the effectiveness of LDCT screening. Most cost-effectiveness analyses compared stage shifts between the screening and non-screening arms to estimate the benefits of early diagnosis^7–9^. While some studies tried to evaluate the lead time in sensitivity analyses, relatively few of them actually quantified the effect of lead-time bias^10^. For those trying to account for lead-time bias, several approaches have been proposed (see Supplementary Table S1). Some studies presumed lung cancer develops rapidly with varying lead times, ranging from zero to two years^7–9,11^. Conventional methods estimated lead time from volume doubling time^12,13^; however, lead time estimation appears more complicated. For instance, lead time could vary according to different pathologies and stages^14^. Although stage-specific lead time was estimated in one study^13^, less advanced cancer appeared to show a longer lead time^15^. Recently, researchers simulated the natural history of different histological subtypes of lung cancer to adjust for lead-time bias^16^, and estimated stage-dependent lead time by comparing the age at screening detection and the age at symptomatic presentation during the same period^17^.

Along the same line, we raised the question from a different angle: The screening, if effective, would generally diagnose cancer at earlier stages and at younger ages, which inherently would result in benefits regarding mortality and/or long-term survival. Thus, in contrast to the conventional approach of multiplying the stage-specific life expectancy (LE) by stage shift for LE gained and subtracting a hypothetical lead time, we proposed measuring the loss-of-LE saved, leveraging the ‘difference-in-differences’ concept from economics. Using Taiwan’s nationwide lung cancer patients, we compared the LE of cancer cases with that of age-, sex-, calendar year-, and major comorbidities-matched referents to estimate the loss-of-LE. By doing so, we adjusted for the effect of more patients diagnosed at earlier stages and younger ages, or different distributions of age, sex, and calendar year due to screening. The pathology- and stage-specific loss-of-LE multiplied by pathology and stage shift would be the loss-of-LE saved from early diagnosis^18^. Moreover, the difference in health benefits estimated by LE gained versus loss-of-LE saved would be the magnitude of lead-time bias that could be reduced or adjusted through this method.

## Materials and methods

We first stratified Taiwan’s nationwide lung cancer patients aged 55–82 by pathology and stage. The survival was extrapolated to lifetime for LE. Second, we randomly selected age-, sex-, calendar year-, and major comorbidities-matched referents from our general population and compared the LE of lung cancer patients with that of the matched referents for loss-of-LE. Third, we multiplied the LE and loss-of-LE by the pathology and stage shift in the National Lung Screening Trial (NLST) for LE gained and loss-of-LE saved^1^. We compared the effectiveness of lung cancer screening measured by LE gained and loss-of-LE saved. Finally, we estimated the LE and loss-of-LE for lung cancer patients who were smokers to explore the potential effect of smoking.

### Survival of patients stratified by pathology and stage

Because eligible participants in the NLST were between 55 and 75 years of age and a maximum follow-up of seven years was used to determine the incidence of lung cancer^1^, we abstracted all pathologically-verified lung cancer patients aged 55–82 during 2002–2015 from the Taiwan National Cancer Registry database for the analysis. We categorized the histopathology into small-cell lung cancer, squamous-cell non-small-cell lung cancer (SqCC), adenocarcinoma, and non-SqCC other than adenocarcinoma. Each patient’s tumor stage was defined according to the classifications provided by the American Joint Committee on Cancer, 6th and 7th editions. Because a completely different categorization of lung adenocarcinoma was proposed in 2011^19,20^, adenocarcinoma in situ (AIS), minimally invasive adenocarcinoma (MIA), and bronchioloalveolar carcinoma (BAC) in stage 0/IA/IB were assigned as three specific subcategories for analysis. Each patient’s identification information was linked to the National Mortality Registry database and followed up from the day of diagnosis until the end of 2017. We used the Kaplan–Meier method to estimate the survival function up to the limit of follow-up.

### Extrapolating the survival to lifetime for life expectancy (LE)

The survival functions of different lung cancer subtypes were extrapolated to lifetime using a rolling extrapolation method^21^, which was carried out in the following four steps: First, by using the life tables in different calendar years, we simulated an age-, sex-matched reference population and estimated the lifetime Kaplan–Meier survival function. Second, we calculated the survival ratio between the lung cancer cohort and reference population at each time point and performed logit transformation of the ratio. Third, we used restricted cubic splines models to fit the logit-transformed relative survival (see Supplementary Fig. S1). Fourth, the first restricted cubic splines model, together with the survival function of the reference population beyond the follow-up limit, was used to extrapolate survival for the next month. Then, with the new end of survival, we fitted the second restricted cubic splines model to extrapolate survival one further month. By repeatedly performing the above procedure month-by-month (*i.e.*, rolling over), we were able to obtain the survival of lung cancer patients over their lifetimes. This method has been shown to produce a relatively accurate estimate of lifetime survival^21^. We used the iSQoL2 statistical package, which can be downloaded at http://sites.stat.sinica.edu.tw/isqol/, for computations.

### Age-, sex-, calendar year-, and comorbidities-matched referents

We interlinked the National Registries for Beneficiaries and Catastrophic Illnesses databases and matched lung cancer patients with referents randomly selected from the general population at a ratio of one-to-ten. The matching criteria included age, sex, calendar year, and major comorbidities at the time of lung cancer diagnosis, which included the following: (1) malignant neoplasms other than skin cancer or in-situ carcinoma; (2) acute cerebrovascular disease, spinal cord injury, and motor neuron disease; (3) end-stage heart failure, chronic pulmonary diseases, and primary neuromuscular diseases, which required ventilation for 21 or more days; (4) end-stage renal disease; (5) cirrhosis of the liver with poorly-controlled ascites, varicose bleeding, or hepatic coma. These catastrophic illnesses would generally cause premature mortality and the diagnoses are credible because patients with any of the above diagnoses can be waived from co-payments. To prevent abuse, the National Health Insurance stipulates two physicians to validate the documents before approval.

### Loss-of-LE for adjustment of lead-time bias

Similarly, we estimated the LE of matched referents by extrapolating referents’ survival to lifetime. Loss-of-LE was defined as the difference between the LE of lung cancer patients and that of matched referents. We also used the iSQoL2 statistical package to carry out these computations. The 95% confidence intervals (CIs) for estimates of LE and loss-of-LE were obtained through 100 bootstraps.

The Taiwan National Lung Screening Program is a single-arm research-based study^22^. Although it contains pathology and stage information of screened lung cancer cases, we were not able to find a comparable control group diagnosed without screening. The pathology and stage shift under Medicare LDCT screening services has not yet been disclosed. Therefore, we borrowed the pathology and stage shift data from the NLST^1^. The LE and loss-of-LE of pathology- and stage-specific lung cancer were multiplied by the pathology and stage shift for LE gained and loss-of-LE saved. To compute the 95% prediction intervals of LE gained and loss-of-LE saved, we created a decision tree (see Supplementary Fig. S2) and simulated 10,000 NLST participants with 1,000 iterations. Consistent with the approach in prior literature for following up lung cancer incidence over 7 years^23^, we assumed all excess lung cancers in the screening arm were over-diagnosed.

### LE and loss-of-LE for lung cancer patients who were smokers

Our National Cancer Registry data included smoking information of lung cancer patients beginning from 2011. They were followed up until the end of 2018. We applied our method to extrapolate the survival function and estimate the LE. The 8-year follow-up period was too short for estimating the lifetime survival functions of age-, sex-, calendar year-, and comorbidities-matched referents accurately. We thus directly compared the survival of lung cancer patients with that of the age-, sex-, calendar year-matched reference population simulated from the life tables (i.e., comorbidities were not matched) for loss-of-LE. We also applied the method to the study cohort for comparison.

### Sensitivity analyses

We conducted sensitivity analysis using lung cancer patients who were smokers. The magnitude of over-diagnosis depends critically on the length of follow-up after the final screening^24^. Notably, the over-diagnosis rate in the NLST became 3% after extending the follow-up to 11.3 years^25^, and the lifetime over-diagnosis rate was predicted to be zero. To have a more appropriate estimation and inference, we performed sensitivity analysis hypothesizing 50%, 3%, and 0% of excess cancers were over-diagnosed. We accounted for the timing of cancer detection in the screening and control arms from the NLST^23^. The test sensitivity of LDCT in the NLST was 93.1%^26^, and could be as low as 59%^27^. We thus assumed test sensitivity to be 80% and 60% for analysis. Besides, we also borrowed the stage shift from the Dutch-Belgian lung cancer screening trial (NELSON)^2^ for sensitivity analysis.

### Validating the extrapolation method

We used the survival data of patients who were diagnosed during the first 8 years and extrapolated their survival up to 16 years. Because these patients were actually followed from 2002 until the end of 2017, the mean survival duration within the 16-year follow-up period using the Kaplan–Meier method was considered as the gold standard. The relative bias was computed by comparing the difference in values between the extrapolation and the Kaplan–Meier estimation. The same validation method was applied to lung cancer patients who were smokers. That is, we used the survival data of patients diagnosed during the first 5 years (2011–2015) and extrapolated them to 8 years (2011–2018), which were then compared with the 8-year Kaplan–Meier estimates for relative biases.

### Ethics approval, consent to participate

The Institutional Review Board of National Cheng Kung University Hospital approved this study before commencement (B-EX-107–050). Consent was waived by the Institutional Review Board. Study methods were performed in accordance with the STROBE guidelines.

## Results

### Comparing life expectancies for loss-of-LE

**Figure 1:**
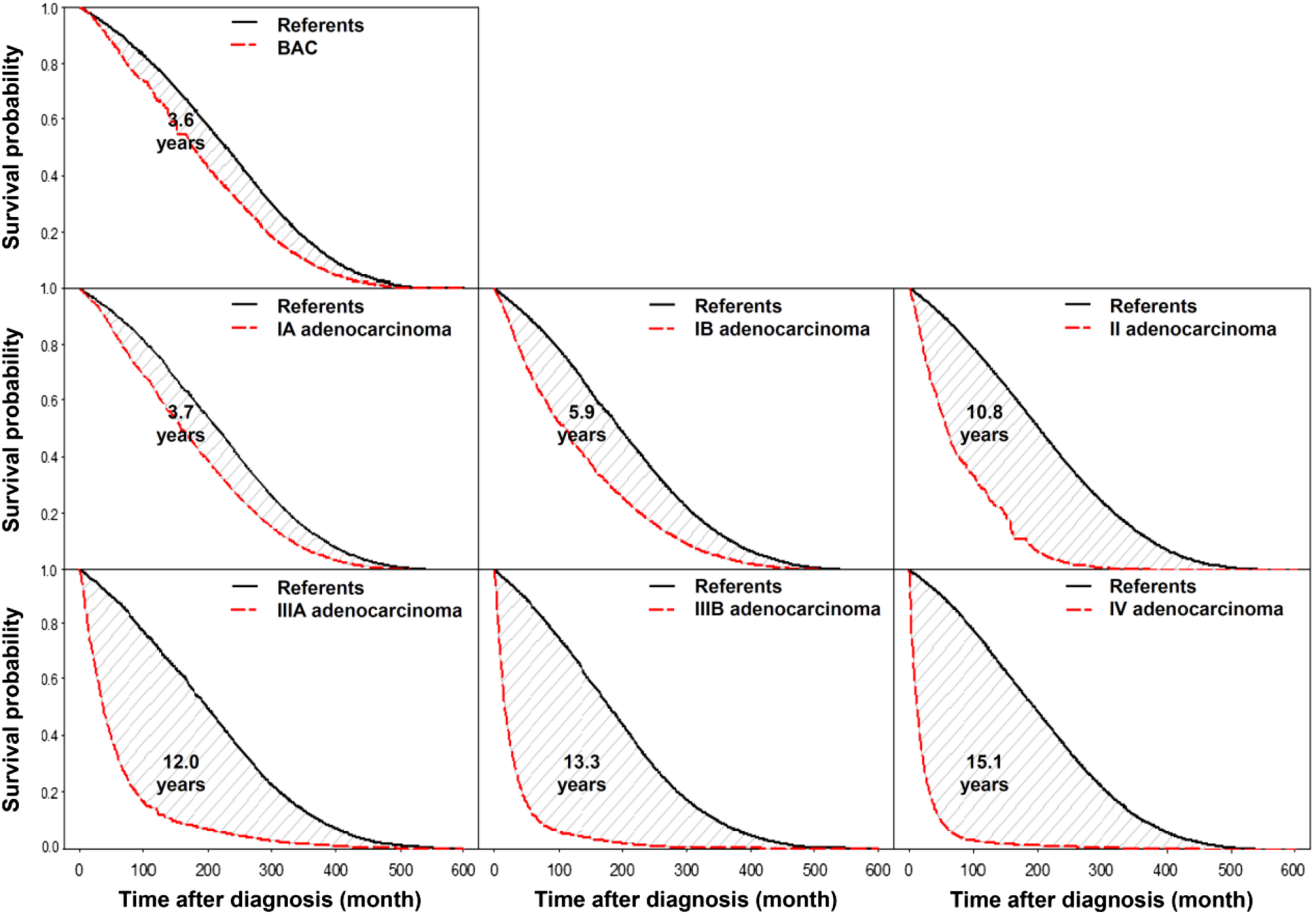
Lifetime survival curves of lung cancer cases and matched referents by stage. The shaded area is the loss-of-life expectancy. *BAC* bronchioloalveolar carcinoma.

During 2002–2015, a total of 78,897 lung cancer patients aged 55–82 were analyzed and one-to-ten matched with 788,820 referents randomly selected from the general population. The clinical characteristics of lung cancer patients and referents were completely matched (Table 1). Table 2 summarizes the LEs of lung cancer patients and matched referents stratified by pathology and stage. Patients with stage IA and stage IV adenocarcinoma were diagnosed at an average age of 66.3 (standard deviation (SD): 7.2) and 68.4 (SD: 7.7), and had a LE of 14.5 (95% CI: 12.6 to 16.4) and 1.9 (95% CI: 1.8 to 2.1) years, respectively. Figure 1 shows the loss-of-LE, which is the difference between the area under the lifetime survival curve of patients and that of matched referents. The loss-of-LEs of patients with stage IA and stage IV adenocarcinoma were 3.7 (95% CI: 1.0 to 5.8) and 15.1 (95% CI: 14.8 to 15.4) years, respectively (Table 2). We found that lung cancer patients who were smokers usually lost 0.5–1 year more in LE than the whole study cohort stratified by pathology and stage.

**Table 1:** Clinical characteristics of lung cancer patients and age-, sex-, year-, comorbidities-matched referents.

		Cumulative number	Male	Calendar years		Catastrophic illness*				
				2002–2010	2011–2015	Cancer	Neural	Respiratory	ESRD	Cirrhosis
			%	%	%	%	%	%	%	%
SCLC	Limited Referents	2176 21,760	90.4	58.9	41.1	6.9	0.1	0.6	0.8	0.1
	Extensive Referents	5156 51,560	90.1	55.1	44.9	6.1	0	0.6	1.0	0.2
SqCC	I Referents	1976 19,720	89.9	58.5	41.5	13.5	0	0.3	1.8	0.3
	II Referents	1255 12,550	92.8	49.3	50.7	10.1	0	0.1	1.0	0.2
	IIIA Referents	2175 21,730	91.1	51.5	48.5	9.7	0.0	0.5	1.4	0.2
	IIIB Referents	3411 34,090	90.8	64.2	35.8	8.1	0.1	0.4	0.9	0.3
	IV Referents	6571 65,700	85.3	55.9	44.1	9.5	0.1	0.4	0.8	0.2
Adenocarcinoma**	BAC Referents	882 8820	34.0	53.2	46.8	12.7	0	0.0	0.9	0
	I Referents	7930 79,290	44.0	37.5	62.5	13.2	0.0	0.1	0.9	0.1
	IA IB	4453 3468	41.8 46.8	32.1 44.4	67.9 55.6	14.8 11.2	0 0.0	0.0 0.1	1.1 0.7	0.0 0.1
	II Referents	1587 15,870	50.1	41.3	58.7	10.1	0	0.1	0.9	0.1
	IIIA Referents	2792 27,920	51.1	47.8	52.2	9.0	0	0.1	0.5	0.1
	IIIB Referents	4398 43,980	58.1	73.2	26.8	6.1	0	0.1	0.8	0.1
	IV Referents	28,109 281,050	51.0	46.8	53.2	5.7	0.0	0.2	0.9	0.1
Other non-SqCC	I Referents	852 8520	69.6	57.9	42.1	11.4	0	0.5	1.8	0.1
	II Referents	410 4100	75.1	49.8	50.2	8.3	0.0	0.2	2.0	0.2
	IIIA Referents	841 8410	71.1	60.3	39.7	9.2	0	0	1.1	0.1
	IIIB Referents	1622 16,220	75.8	74.5	25.5	6.4	0.1	0.4	0.6	0.1
	IV Referents	6754 67,530	67.7	64.8	35.2	6.3	0.0	0.4	1.2	0.2
All Referents		78,897 788,820	63.9	52.5	47.5	7.8	0.0	0.3	0.9	0.1

*BAC* bronchioloalveolar carcinoma, *ESRD* end-stage renal disease, *SCLC* small-cell lung cancer, *SqCC* squamous-cell non-small-cell lung cancer.

*Selected major comorbidities include: 1. malignant neoplasms other than skin cancer or in-situ carcinoma; 2. acute cerebrovascular disease, spinal cord injury, and motor neuron disease; 3. end-stage heart failure, chronic pulmonary diseases, and primary neuromuscular diseases, which required ventilation for 21 or more days; 4. end-stage renal disease; 5. cirrhosis of liver with poorly-controlled ascites, varicose bleeding, or hepatic coma.

**Adenocarcinoma in situ (*n* = 254) and minimally-invasive adenocarcinoma (*n* = 102) were not analyzed due to small sample sizes and high censored rates.

**Table 2:** Life expectancy (LE) and loss-of-LE of the study cohort and lung cancer patients who were smokers.

Pathology	Stage		Number	Age at diagnosis	Life expectancy (LE)	Loss-of-LE by comparing LE with that of age-, sex-, and calendar year- matched reference population*	Loss-of-LE by comparing LE with that of age-, sex-, calendar year-, and comorbidities-matched referents
				Mean (SD)	Life-years (95% CI)	Life-years (95% CI)	Life-years (95% CI)
SCLC	Limited	Study cohort	2176	69.2 (7.4)	2.3 (2.0 to 2.7)	13.2 (12.6 to 13.8)	12.5 (11.5 to 13.4)
		Smokers	1042	68.0 (7.3)	2.6 (2.0 to 3.2)	13.8 (13.2 to 14.4)	
	Extensive	Study cohort	5156	69.7 (7.3)	0.8 (0.7 to 0.9)	14.4 (14.2 to 14.6)	14.2 (13.6 to 14.8)
		Smokers	2559	68.4 (7.2)	0.8 (0.7 to 0.9)	15.5 (15.3 to 15.7)	
SqCC	I	Study cohort	1976	70.5 (7.0)	7.4 (6.7 to 8.1)	7.2 (6.2 to 8.2)	6.4 (5.3 to 7.2)
		Smokers	893	70.1 (6.9)	6.8 (5.0 to 8.6)	8.1 (6.3 to 9.9)	
	II	Study cohort	1255	70.1 (7.0)	5.3 (4.7 to 6.6)	9.5 (8.7 to 10.3)	8.7 (7.5 to 9.7)
		Smokers	656	69.7 (7.1)	5.7 (4.3 to 7.1)	9.5 (8.1 to 10.9)	
	IIIA	Study cohort	2175	70.5 (7.2)	2.6 (2.4 to 3.0)	11.8 (11.2 to 12.4)	11.8 (11.3 to 12.3)
		Smokers	1033	70.0 (7.3)	2.6 (2.2 to 3.0)	12.4 (11.8 to 13.0)	
	IIIB	Study cohort	3411	70.1 (7.2)	1.8 (1.6 to 2.1)	13.1 (12.7 to 13.5)	12.2 (11.0 to 13.2)
		Smokers	1249	69.3 (7.4)	1.6 (1.4 to 1.8)	14.0 (13.6 to 14.4)	
	IV	Study cohort	6571	70.3 (7.3)	1.0 (0.9 to 1.1)	13.9 (13.7 to 14.1)	13.1 (12.7 to 13.6)
		Smokers	2700	69.5 (7.3)	0.8 (0.7 to 0.9)	14.5 (14.3 to 14.7)	
Adenocarcinoma	I	Study cohort	7930	67.0 (7.3)	13.6 (11.7 to 14.4)	5.2 (4.0 to 6.4)	4.0 (2.9 to 6.3)
		IA	4453	66.3 (7.2)	14.5 (12.6 to 16.4)		3.7 (1.0 to 5.8)
		IB	3468	67.8 (7.3)	11.2 (9.9 to 12.7)		5.9 (3.9 to 7.6)
		Smokers	1450	67.0 (7.1)	11.0 (8.1 to 13.9)	6.3 (3.4 to 9.2)	
	II	Study cohort	1587	67.6 (7.4)	6.6 (6.1 to 8.0)	11.4 (10.4 to 12.4)	10.8 (9.1 to 11.3)
		Smokers	385	67.6 (7.5)	6.9 (4.7 to 9.1)	10.0 (7.6 to 12.4)	
	IIIA	Study cohort	2792	68.0 (7.6)	5.2 (4.5 to 6.1)	12.4 (11.6 to 13.2)	12.0 (10.8 to 13.4)
		Smokers	640	67.6 (7.5)	4.8 (3.8 to 5.8)	12.1 (10.9 to 13.3)	
	IIIB	Study cohort	4398	69.2 (7.5)	2.6 (2.4 to 2.8)	13.8 (13.4 to 14.2)	13.3 (12.8 to 14.0)
		Smokers	744	67.6 (7.7)	2.5 (2.1 to 2.9)	14.3 (13.7 to 14.9)	
	IV	Study cohort	28,109	68.4 (7.7)	1.9 (1.8 to 2.1)	15.4 (15.2 to 15.6)	15.1 (14.8 to 15.4)
		Smokers	6869	67.8 (7.7)	1.5 (1.3 to 1.7)	15.3 (15.1 to 15.5)	
Other non-SqCC	I	Study cohort	852	69.6 (7.6)	9.3 (8.1 to 10.3)	6.5 (4.9 to 8.1)	5.4 (4.2 to 7.5)
		Smokers	232	68.4 (7.2)	11.2 (7.7 to 14.7)	5.0 (1.7 to 8.3)	
	II	Study cohort	410	69.5 (7.8)	4.4 (3.6 to 6.4)	11.4 (10.0 to 12.8)	10.7 (8.4 to 12.0)
		Smokers	139	68.7 (7.8)	3.9 (1.7 to 6.1)	12.0 (9.6 to 14.4)	
	IIIA	Study cohort	841	70.1 (7.5)	3.1 (2.6 to 4.0)	12.3 (11.5 to 13.1)	11.7 (10.2 to 13.2)
		Smokers	203	69.3 (7.2)	3.3 (1.9 to 4.7)	12.4 (10.8 to 14.0)	
	IIIB	Study cohort	1622	70.3 (7.6)	1.8 (1.5 to 2.1)	13.4 (13.0 to 13.8)	13.4 (12.6 to 14.3)
		Smokers	296	69.4 (7.8)	1.6 (0.8 to 2.4)	13.9 (12.7 to 15.1)	
	IV	Study cohort	6754	70.5 (7.4)	1.0 (0.9 to 1.1)	14.1 (13.9 to 14.3)	14.5 (14.0 to 15.0)
		Smokers	1469	69.7 (7.7)	0.7 (0.6 to 0.8)	14.7 (14.3 to 15.1)	

*SCLC* small-cell lung cancer, *SqCC* squamous-cell non-small-cell lung cancer.

* Smoking information of lung cancer patients was available from 2011, the follow-up period was too short to estimate the lifetime survival functions of age-, sex-, calendar year-, and comorbidities-matched referents accurately. Survival of lung cancer patients was directly compared with that of age-, sex-, calendar year-matched reference population simulated from the life tables for loss-of-LE. We also applied the method to the study cohort for comparison.

### Adjustment of age, sex, and calendar year to reduce lead-time bias

**Figure 2:**
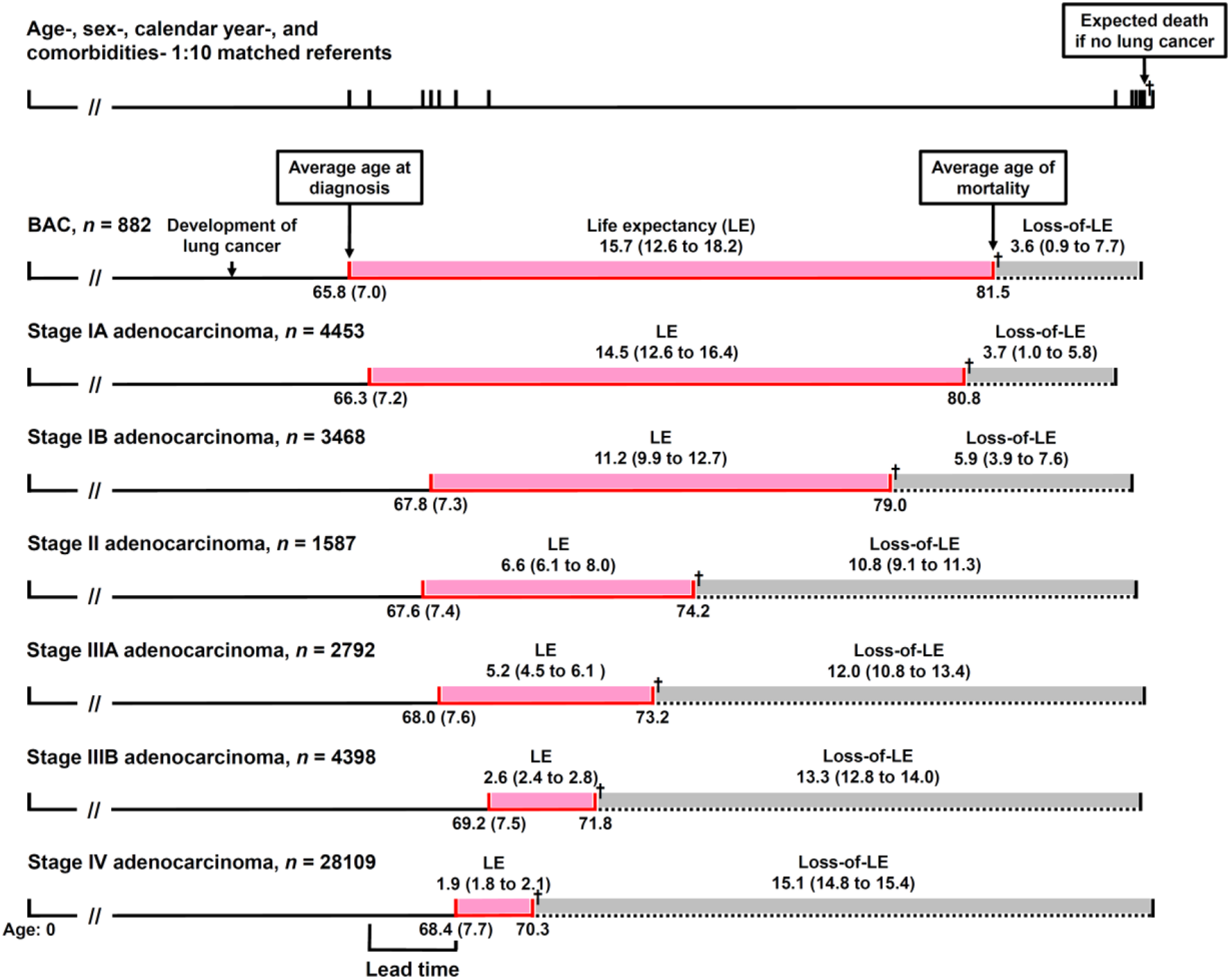
Adjustment of age, sex, calendar year, and comorbidities at diagnosis for lead-time bias. A patient with stage IV adenocarcinoma is routinely diagnosed at a mean age of 68.4 (see Table 2). If the patient was diagnosed earlier at stage IA (at a mean age of 66.3), the average gain in life expectancy (LE) would be 14.5–1.9 = 12.6 years. However, if we take different age, sex, year of diagnosis, and comorbidities into consideration and compared the loss-of-LE, the average savings of loss-of-LE would be 15.1–3.7 = 11.4 years, which implies an adjustment for lead-time bias. The values in parentheses for age and LE/loss-of-LE denote the standard deviations and 95% confidence intervals, respectively. † denotes mortality. *BAC* bronchioloalveolar carcinoma.

Figure 2 illustrates how lead-time bias could be adjusted by using loss-of-LE. According to Table 2, a patient with stage IV adenocarcinoma was generally diagnosed at a mean age of 68.4 and had a mean LE of 1.9 years. If the patient was diagnosed earlier at stage IA (at a mean age of 66.3 and with a mean LE of 14.5 years), the average gain in LE would be 12.6 years. However, if we took different age, sex, calendar year of diagnosis, and major comorbidities into consideration and compared the loss-of-LEs, the average savings of loss-of-LE would be 11.4 (= 15.1–3.7) years. In other words, our proposed method of comparing loss-of-LEs, or measuring difference-in-differences, adjusts for the overestimation of LE gained resulting from early diagnosis of lung cancer, and the magnitude was 1.2 (= 12.6–11.4) years.

**Figure 3:**
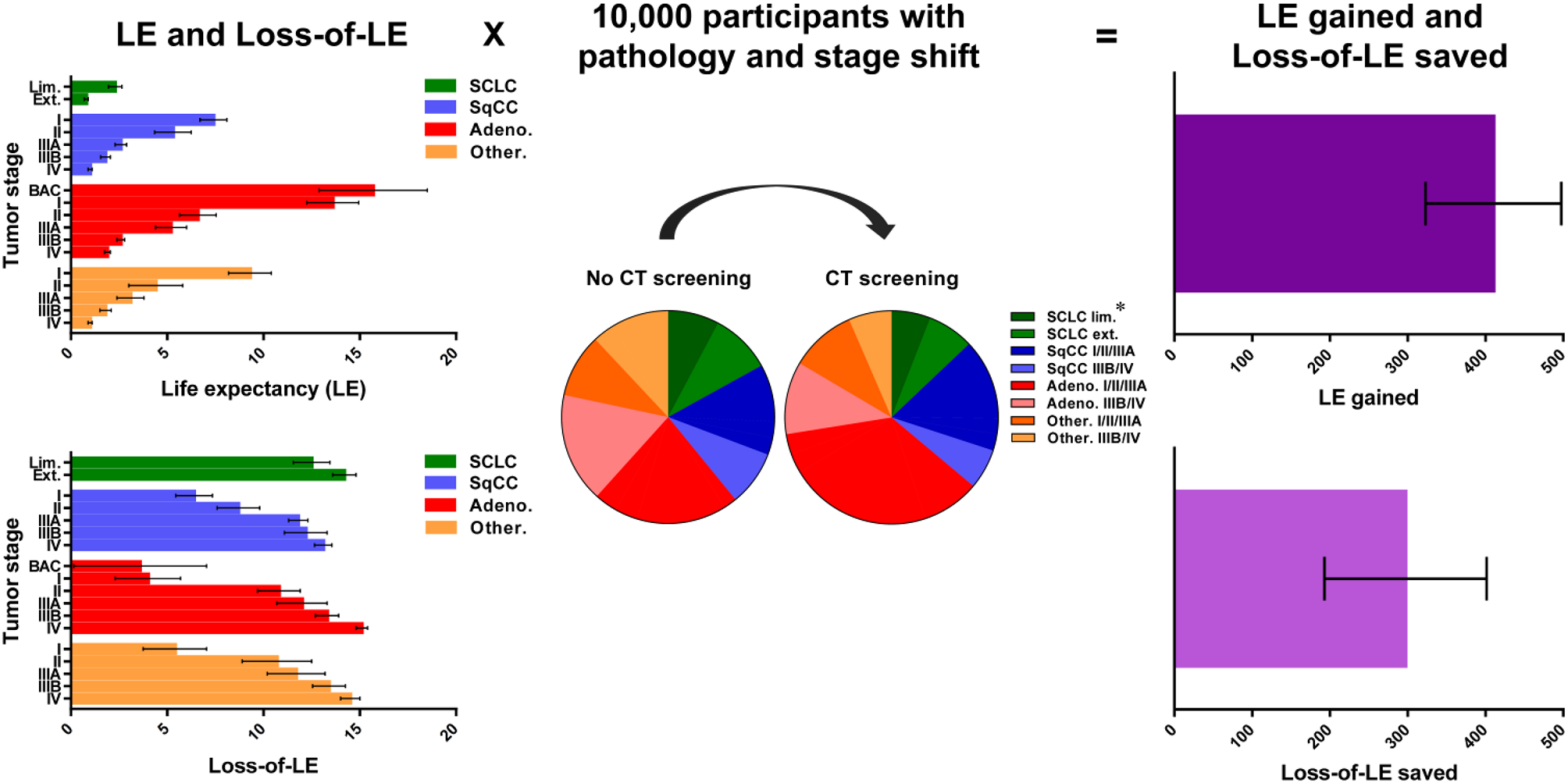
Multiplying LE and loss-of-LE by the pathology and stage shift of 10,000 NLST participants for LE gained and loss-of-LE saved. The error bars denote the 95% confidence intervals of LE/loss-of-LE and the 95% prediction intervals of LE gained/loss-of-LE saved. *98% and 97% in the control and screening arms, respectively, for which the pathology and stage of lung cancer were known. *Adeno* adenocarcinoma, *BAC* bronchioloalveolar carcinoma, *CT* computed tomography, *Ext*. extensive stage, *Lim*. limited stage, *NLST *National Lung Screening Trial, *Other*. non-SqCC other than adenocarcinoma, *SCLC *small-cell lung cancer, *SqCC *squamous-cell non-small-cell lung cancer.

Applying such estimations on results of 10,000 participants with the same pathology and stage shift in the NLST and assuming 100% excess lung cancers were over-diagnosed, we compared the effectiveness by measuring LE gained and loss-of-LE saved (Fig. 3, also see Supplementary Table S2 for details). The benefit of screening using LE gained would be 410.3 (95% prediction interval: 328.4 to 503.3) years. It became 297.1 (95% prediction interval: 187.8 to 396.4) years when using loss-of-LE saved, indicating the former approach would over-estimate the effectiveness by 38%.

### Sensitivity analyses

Table 3 displays that the LE gained and loss-of-LE saved of smokers became lower after weighting the stage shift. Sensitivity analysis hypothesizing 50%, 3%, and 0% of excess cancers were over-diagnosed was performed. We found that the differences between LE gained and loss-of-LE saved slightly increased when the over-diagnosis rate decreased in lung cancer. Sensitivity analyses assuming the test sensitivity of LDCT ranged from 60 to 80%, and stage shift from the NELSON are also shown. All estimates using LE gained were higher than those using loss-of-LE saved.

**Table 3:** One-way sensitivity analyses.

	Incremental effectiveness	
	LE gained	Loss-of-LE saved
	life-years (95% prediction interval)	life-years (95% prediction interval)
**Smoking**		
Study cohort	410.3 (328.4 to 503.3)	297.1 (187.8 to 396.4)
Smokers	354.5 (237.9 to 474.9)	244.4 (115.8 to 369.7)*
**Proportion of excess cancers over-diagnosed**		
100%	410.3 (328.4 to 503.3)	297.1 (187.8 to 396.4)
50%	635.2 (545.3 to 728.6)	515.0 (407.4 to 622.4)
3%	863.7 (768.8 to 962.4)	738.8 (625.2 to 852.2)
0%	879.2 (783.9 to 978.2)	753.9 (639.8 to 867.7)
**Test sensitivity of LDCT**		
93.1%^26^	410.3 (328.4 to 503.3)	297.1 (187.8 to 396.4)
80%	299.8 (227.1 to 375.3)	203.9 (116.9 to 290.7)
60%	132.2 (77.7 to 188.9)	59.8 (-5.5 to 124.9)
**Stage shift**		
NLST^1^	410.3 (328.4 to 503.3)	297.1 (187.8 to 396.4)
NELSON^2^	536.3 (459.3 to 620.6)	429.9 (331.3 to 523.7)

*LE* life expectancy, *LDCT* low-dose computed tomography, *NELSON* Dutch-Belgian lung cancer screening trial, *NLST* National Lung Screening Trial.

*Smoking information of lung cancer patients was available from 2011, the follow-up period was too short to estimate the lifetime survival functions of age-, sex-, calendar year-, and comorbidities-matched referents accurately. Survival of lung cancer patients was directly compared with that of age-, sex-, calendar year-matched reference population simulated from the life tables for loss-of-LE.

### Validation of the extrapolation method

There were 34,427 patients diagnosed during the first 8 years, between 2002 and 2009, of which the survival curves were extrapolated to 2017 and compared with the Kaplan–Meier estimates based on the 16-year follow-up (Supplementary Table S3). The relative biases of the extrapolation ranged between 1.1% and 7.1%. The results of lung cancer patients who were smokers were also similar.

## Discussion

Although a randomized screening trial beginning follow-up from randomization would eliminate lead-time bias^28^, detailed trial data might not be available for researchers. In contrast, research using observational real-world data is subject to lead-time bias. For example, cases detected through an effective national screening program usually show increased proportions of cases in early stages, and their age would be younger than those who are not diagnosed through screening, indicating the existence of lead time. We proposed estimating the loss-of-LE saved to adjust for different distributions of age, sex, and calendar year at early diagnosis to reduce this bias, and we have the following arguments to support the above assertion: First, the validation of our extrapolation method using half period (*i.e.*, 8 years) of survival showed that the relative biases were all less than 7.1% (Supplementary Table S3). The month-by-month rolling over algorithm combined with the well-fit model of restricted cubic splines (Supplementary Fig. S1) for the full period of survival also assure the accurate estimate of LE^21^. As our follow-up of 16 years was generally longer than the usual LE of lung cancer patients, the validity of extrapolated results would be acceptable. Second, since the referents were selected by matching every case by age, sex, calendar year of diagnosis, and major comorbidities (Table 1), the difference between the LE of pathology- and stage-specific cancer patients and that of matched referents could mainly be attributed to the occurrence of lung cancer. That is, there would be little confounding on all estimates of loss-of-LE (Table 2). Third, as the weighted sums of loss-of-LE in screening and control arms have already accounted for the different pathologies and stage distributions of lung cancer patients (Supplementary Table S2), comparison of them for loss-of-LE saved has adjusted for the stage shift and excess incidence of lung cancer.

In real-world practice, it is challenging to select a matched control from the beginning to observe survival for adjustment of lead-time bias. Our method might provide an alternative solution for this issue. However, even though the Centers for Medicare & Medicaid Services initiated reimbursement for LDCT screening in February 2015^4^, real-world pathology and stage shift has not yet been disclosed. We therefore tentatively borrowed the pathology and stage shift in the NLST^1^ and simulated 10,000 participants to demonstrate the calculations of LE gained and loss-of-LE saved (Fig. 3). Compared with an incremental LE of 316 life-years per 10,000 participants in an epoch-making cost-effectiveness study^28^, the loss-of-LE saved in our study, equal to 297.1 life-years per 10,000 participants is slightly more conservative. Once the real-world pathology and stage shift becomes available, our method could directly be applied to provide a quick evaluation of the effectiveness of LDCT screening.

It is interesting to note that cases with BAC and stage IA adenocarcinoma were diagnosed at a younger age than late-staged patients (Fig. 2). The difference between the ages of diagnosis at early and late stages could be regarded as an approximate estimate of lead time with the same pathology, assuming that people with the same age, sex, and calendar year of diagnosis would be of the same risk set. Further matching performed on major comorbidities would improve the validity of this assumption. However, because of the higher cell proliferation rates^29^ and shorter doubling times^30,31^ of small-cell lung cancer (SCLC) and SqCC than those of adenocarcinoma, patients with these subtypes of cancers had a shorter preclinical phase. The differences in age distribution between early- and late-stage lung cancers of such subtypes were smaller. Consequently, our proposed method would be less useful. For example, patients of extensive stage SCLC and stage IV SqCC had mean LEs of 0.8 and 1.0 years, respectively (Table 2). If the patients were diagnosed earlier at limited stage for SCLC and stage I for SqCC with mean LEs of 2.3 and 7.4 years, the mean LEs gained would be 1.5 and 6.4 years, respectively. If we compared the loss-of-LEs, the mean loss-of-LEs saved would be 1.7 (= 14.2–12.5) and 6.7 (= 13.1–6.4) years, respectively, which were similar to the LEs gained. In other words, our proposed method of estimating loss-of-LE saved would not be applicable in lung cancers with rapid proliferation rates, which offers a short or minimal preclinical phase for early detection.

Several limitations must be acknowledged in this study. First, we used a diagnosed lung cancer cohort from Taiwan’s nationwide database instead of cases detected from a screening program. The results might not be applicable to settings in other countries. Besides, most of the diagnoses were made through symptoms instead of screening, which could underestimate the lead time. However, the early-stage patients in our study had higher proportions of other malignancies upon diagnosis than late-stage ones (Table 1), which might imply that some of the early-stage patients were diagnosed through active surveillance. Therefore, the magnitude of underestimation would not be overly large. Second, due to the lack of information on smoking among the reference population, we did not directly account for the difference in exposure to smoking while extrapolating the survival for LE and matching the comorbidities of referents for loss-of-LE. However, sensitivity analysis using lung cancer patients who were smokers was conducted (Table 3). The LE gained and loss-of-LE saved of smokers were lower than those of the study cohort. Third, we hypothesized 100% of excess lung cancers were over-diagnosed and provided no details on the pathology and stage of the cases considered to be over-diagnosed. Over-diagnosis denotes detection of lung cancer which will not go on to cause symptoms or mortality before death from other causes^32^. That is, death from other causes preceding lung cancer death, or no loss-of-LE, is a prerequisite for over-diagnosis. Our method for estimating loss-of-LE saved might have indirectly adjusted for this bias. We assumed 50%, 3%, and 0% of excess cancers to be over-diagnosed in the sensitivity analyses (Table 3), which might have considered the influence of the length of follow-up period on over-diagnosis. AIS and MIA have been associated with an increased risk of over-diagnosis^33^. However, they became new categories of lung cancer starting from 2011^19,20^. Given the small sample sizes and high censored rates, the loss-of-LEs could not be estimated. No inferior survival for AIS and MIA cases compared to that of referents was observed during the period (Supplementary Fig. S3).

In conclusion, estimating LE gained to evaluate the effectiveness of early diagnosis of lung cancer is subject to lead-time bias. Our approach of multiplying the loss-of-LE by pathology and stage shift to estimate the loss-of-LE saved could adjust for different distributions of age, sex, and calendar year at early diagnosis and reduce this bias.

## Supplementary Information

Supplementary Information.

## Data Availability

The datasets generated and/or analyzed during this study are not publicly available due to confidentiality reasons, but the sufficiently aggregated data used for analyses may be available from the corresponding author upon reasonable request.
