# Supplementary material for: Considering lead-time bias in evaluating the effectiveness of lung cancer screening with real-world data
Source: Sci Rep. 2021 Jun 9;11:12180. doi: 10.1038/s41598-021-91852-6 (PMC8190256; doi:10.1038/s41598-021-91852-6)
Supplement: Supplementary file 1 — Supplementary Information. [file 41598_2021_91852_MOESM1_ESM.doc]

Supplementary information

**Considering lead-time bias in evaluating the effectiveness of lung cancer screening with real-world data**

Running title: Lead-time bias in lung cancer screening

Szu-Chun Yang^1,2^, Jung-Der Wang^3^*, Shi-Yi Wang^2,4^

^1^Department of Internal Medicine, National Cheng Kung University Hospital, College of Medicine, National Cheng Kung University, Tainan, Taiwan. ^2^Department of Chronic Disease Epidemiology, Yale University School of Public Health, New Haven, Connecticut, USA. ^3^Department of Public Health, College of Medicine, National Cheng Kung University, Tainan, Taiwan. ^4^Cancer Outcomes, Public Policy, and Effectiveness Research Center, Yale University School of Medicine, New Haven, Connecticut, USA.

*College of Medicine, National Cheng Kung University, 1 University Road, Tainan 701, Taiwan. telephone: +886-6-2353535 ext 5600, email: [jdwang121@gmail.com](mailto:jdwang121@gmail.com)

E-mail addresses: yangszuchun@gmail.com (S.C.Y.), jdwang121@gmail.com (J.D.W.), shiyi.wang@yale.edu (S.Y.W.).

**Figure S1.** Restricted cubic splines model to fit the logit-transformed relative survival and month-by-month extrapolation to lifetime. Logit-transformed relative survival often decreases sharply in the first few months and gradually approaches a straight line after some time. A cubic spline function with five knots fitted the data scattered around the logit-transformed relative survival curve. All these figures simply show the fitted model for the extrapolation of the first month after the end of follow-up, and the process would be repeatedly performed for every month henceforth until all became deceased. AIS—adenocarcinoma in situ; BAC—bronchioloalveolar carcinoma.


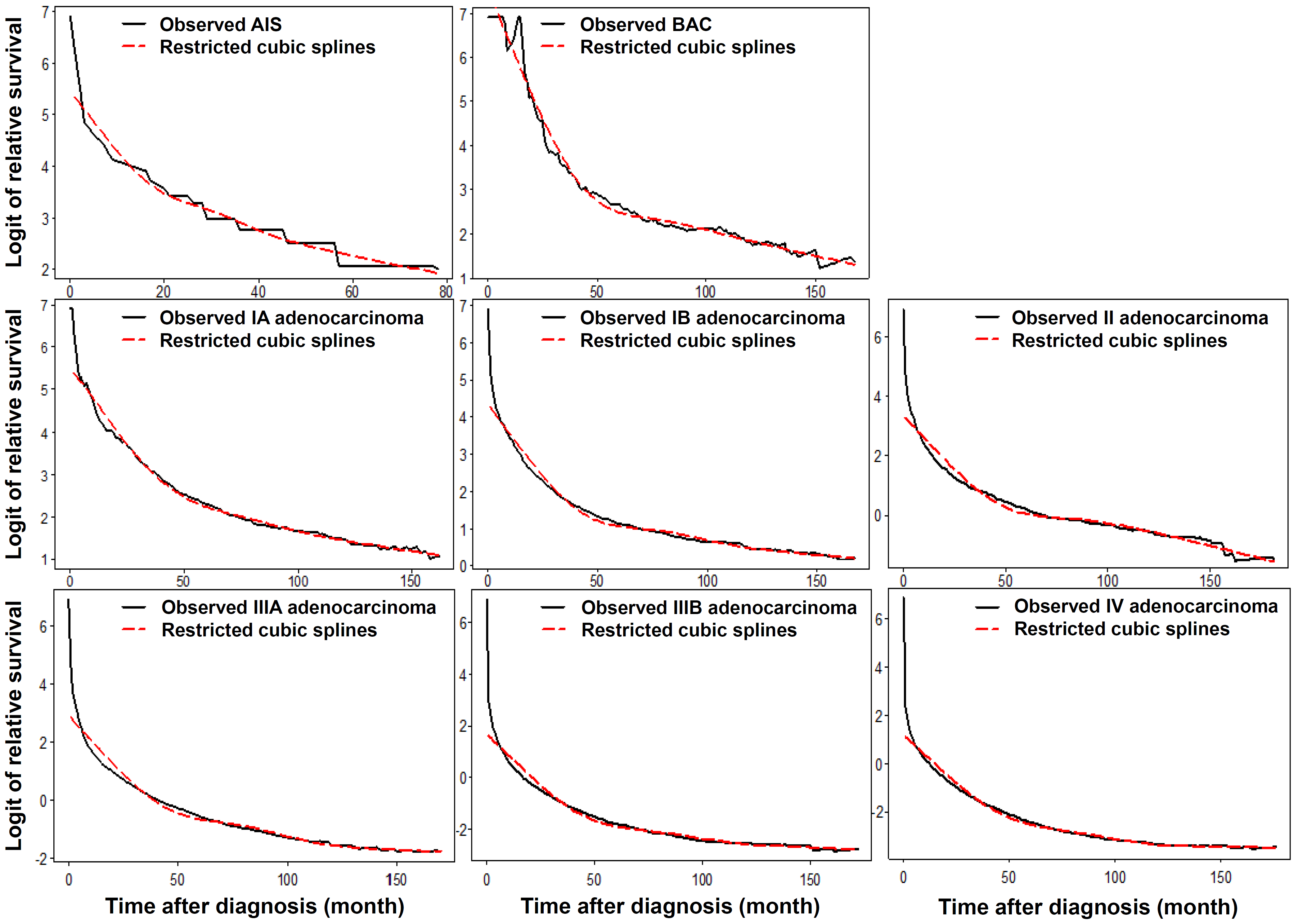


**Figure S2.** Decision tree analysis for the 95% prediction intervals of life expectancy (LE) gained and loss-of-LE saved. Pathology and stage-specific LE and loss-of-LE were assumed to be normally-distributed. BAC—bronchioloalveolar carcinoma; SCLC—small-cell lung cancer; SqCC—squamous-cell lung cancer.

**
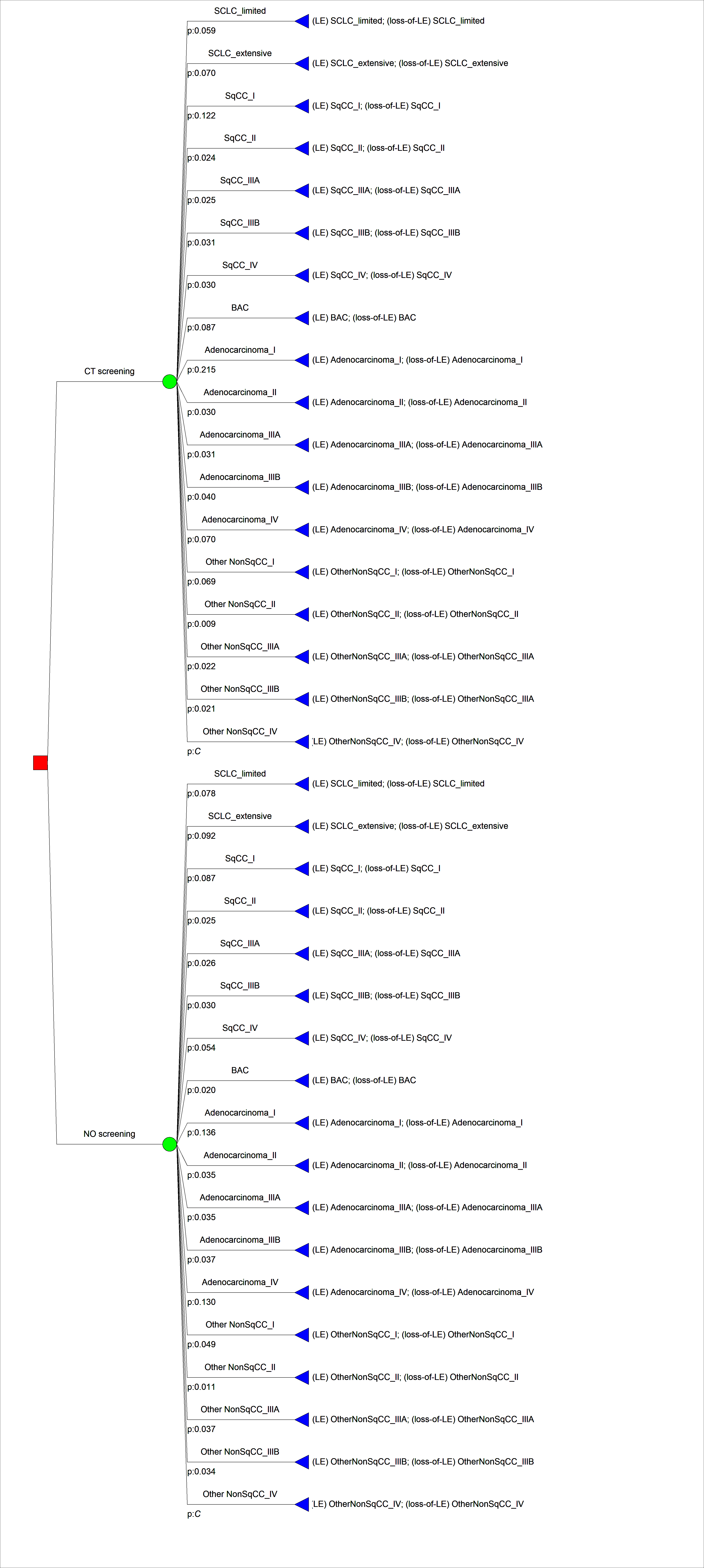
**

**Figure S3.** Comparison of the survival of AIS and MIA cases with that of matched referents. *P* value was calculated using log-rank test for comparison of survival curves. AIS—adenocarcinoma in situ; MIA—minimally-invasive adenocarcinoma.

| **Table S1.** Lead time estimations for lung cancer screening | | |
| --- | --- | --- |
| Author | Year | Lead time estimation |
| Point estimates of lead time: | | |
| Shmueli^7^ | 2013 | Base-case value of lead time was set to 2 years, sensitivity analysis covered the values of 0 and 4 years. |
| Villanti^8^ | 2013 | A 2-year lead time was assumed. |
| Pyenson^9^ | 2014 | A lead time equal to zero was assumed to produce life-years saved figures; however, for costs, a 3-year lead time was assumed (e.g., the SEER cancer incidence for age 75 years was applied to age 72 years). |
| Goldberg^11^ | 2010 | A lead time of 1 year between stage IA and stages IIIA, IIIB, and 2 years between stages IA and IV were assumed. |
| Reich^12^ | 2008 | With a mean volume doubling time of 1 year, 3 years are required for a tumor diameter to increase from 0.5 to 1 cm. Because 1 cm tumors are frequently obscured in chest X-rays, computed tomography would increase survival of stage I adenocarcinomas by approximately 3 years because of lead-time bias. |
| Lead time varied by different pathologies and stages: | | |
| Wisnivesky^13^ | 2003 | Estimates based on expert opinion and doubling time: stage I: 1.5 years; stage II: 2.5 years; stage IIIA: 3.5 years; stage IIIB: 4.0 years; stage IV: 4.5 years. |
| Manser^15^ | 2005 | For more aggressive cancers, the lead time would be short, but for slow growing tumors the lead time could be several years. A lead time of 12 month was assumed. |
| de Koning^16^ | 2014 | Simulating the natural history of different histological subtypes of lung cancer to adjust for lead-time bias. |
| Field^17^ | 2016 | Estimating lead time by comparing the mean ages at detection of the cancers by stage and the ages of symptomatic presentation. The integer age differences amounted to 3 years, 2 years and 1 year, for stages 1–3, respectively. |

**References:**

7 Shmueli, A. *et al.* Cost-effectiveness of baseline low-dose computed tomography screening for lung cancer: the Israeli experience. *Value Health* **16**, 922-931, doi:10.1016/j.jval.2013.05.007 (2013).

8 Villanti, A. C., Jiang, Y., Abrams, D. B. & Pyenson, B. S. A cost-utility analysis of lung cancer screening and the additional benefits of incorporating smoking cessation interventions. *PLoS One* **8**, e71379, doi:10.1371/journal.pone.0071379 (2013).

9 Pyenson, B. S., Henschke, C. I., Yankelevitz, D. F., Yip, R. & Dec, E. Offering lung cancer screening to high-risk Medicare beneficiaries saves lives and is cost-effective: an actuarial analysis. *Am Health Drug Benefits* **7**, 272-282 (2014).

11 Goldberg, S. W., Mulshine, J. L., Hagstrom, D. & Pyenson, B. S. An actuarial approach to comparing early stage and late stage lung cancer mortality and survival. *Population Health Management* **13**, 33-46, doi:10.1089/pop.2009.0010 (2010).

12 Reich, J. M. A critical appraisal of overdiagnosis: estimates of its magnitude and implications for lung cancer screening. *Thorax* **63**, 377-383, doi:10.1136/thx.2007.079673 (2008).

13 Wisnivesky, J. P., Mushlin, A. I., Sicherman, N. & Henschke, C. The cost-effectiveness of low-dose CT screening for lung cancer: preliminary results of baseline screening. *Chest* **124**, 614-621, doi:10.1378/chest.124.2.614 (2003).

15 Manser, R. *et al.* Cost-effectiveness analysis of screening for lung cancer with low dose spiral CT (computed tomography) in the Australian setting. *Lung Cancer* **48**, 171-185, doi:10.1016/j.lungcan.2004.11.001 (2005).

16 de Koning, H. J. *et al.* Benefits and harms of computed tomography lung cancer screening strategies: a comparative modeling study for the U.S. Preventive Services Task Force. *Ann Intern Med* **160**, 311-320, doi:10.7326/m13-2316 (2014).

17 Field, J. K. *et al.* The UK Lung Cancer Screening Trial: a pilot randomised controlled trial of low-dose computed tomography screening for the early detection of lung cancer. *Health Technol Assess* **20**, 1-146, doi:10.3310/hta20400 (2016).

| **Table S2.** Multiplying LE and loss-of-LE by the pathology and stage shift of 10,000 NLST participants for LE gained and loss-of-LE saved. | | | | | | | | |
| --- | --- | --- | --- | --- | --- | --- | --- | --- |
|  | | LE and Loss-of-LE | | No CT screening | | CT screening** | | Incremental effectiveness |
|  |  | LE | Loss-of-LE | Distribution | Outcome | Distribution | Outcome |  |
|  |  | *life-years (95% CI)* | *life-years (95% CI)* |  | *life-years*  *(95% prediction interval)* |  | *life-years*  *(95% prediction interval)* | *life-years*  *(95% prediction interval)* |
| Participants with lung cancer* | |  |  | *n* = 352 | Weighted sum of LE  = 1712.8  (1632.9 to 1789.8)  Weighted sum of  loss-of-LE  = 3802.9  (3694.6 to 3911.0) | *n* = 352 | Weighted sum of LE  = 2123.0  (1975.4 to 2274.0)  Weighted sum of  loss-of-LE  = 3505.7  (3330.0 to 3693.6) | LE gained  = 410.3  (328.4 to 503.3)  Loss-of-LE saved  = 297.1  (187.8 to 396.4) |
| SCLC | Limited | 2.3 (2.0 to 2.7) | 12.5 (11.5 to 13.4) | 7.8% |  | 5.9% |  |  |
|  | Extensive | 0.8 (0.7 to 0.9) | 14.2 (13.6 to 14.8) | 9.2% |  | 7.0% |  |  |
| SqCC | I | 7.4 (6.7 to 8.1) | 6.4 (5.3 to 7.2) | 8.7% |  | 12.2% |  |  |
|  | II | 5.3 (4.7 to 6.6) | 8.7 (7.5 to 9.7) | 2.5% |  | 2.4% |  |  |
|  | IIIA | 2.6 (2.4 to 3.0) | 11.8 (11.3 to 12.3) | 2.6% |  | 2.5% |  |  |
|  | IIIB | 1.8 (1.6 to 2.1) | 12.2 (11.0 to 13.2) | 3.0% |  | 3.1% |  |  |
|  | IV | 1.0 (0.9 to 1.1) | 13.1 (12.7 to 13.6) | 5.4% |  | 3.0% |  |  |
| Adenocarcinoma | BAC | 15.7 (12.6 to 18.2) | 3.6 (0.9 to 7.7) | 2.0% |  | 8.7% |  |  |
|  | I | 13.6 (11.7 to 14.4) | 4.0 (2.9 to 6.3) | 13.6% |  | 21.5% |  |  |
|  | II | 6.6 (6.1 to 8.0) | 10.8 (9.1 to 11.3) | 3.5% |  | 3.0% |  |  |
|  | IIIA | 5.2 (4.5 to 6.1) | 12.0 (10.8 to 13.4) | 3.5% |  | 3.1% |  |  |
|  | IIIB | 2.6 (2.4 to 2.8) | 13.3 (12.8 to 14.0) | 3.7% |  | 4.0% |  |  |
|  | IV | 1.9 (1.8 to 2.1) | 15.1 (14.8 to 15.4) | 13.0% |  | 7.0% |  |  |
| Other non-SqCC | I | 9.3 (8.1 to 10.3) | 5.4 (4.2 to 7.5) | 4.9% |  | 6.9% |  |  |
|  | II | 4.4 (3.6 to 6.4) | 10.7 (8.4 to 12.0) | 1.1% |  | 0.9% |  |  |
|  | IIIA | 3.1 (2.6 to 4.0) | 11.7 (10.2 to 13.2) | 3.7% |  | 2.2% |  |  |
|  | IIIB | 1.8 (1.5 to 2.1) | 13.4 (12.6 to 14.3) | 3.4% |  | 2.1% |  |  |
|  | IV | 1.0 (0.9 to 1.1) | 14.5 (14.0 to 15.0) | 8.6% |  | 4.4% |  |  |
| BAC—bronchioloalveolar carcinoma; LE—life expectancy; NLST—National Lung Screening Trial; SCLC—small-cell lung cancer; SqCC—squamous-cell non-small-cell lung cancer. *98% and 97% in the control and screening arms, respectively, for which the pathology and stage of lung cancer were known. **Assuming 100% excess cancers (*n* = 45) were over-diagnosed and adjusting for life-years difference during the 7-year time frame. | | | | | | | | |

| **Table S3.** Validation of the extrapolated estimates | | | | | | | | | |
| --- | --- | --- | --- | --- | --- | --- | --- | --- | --- |
|  | | Study cohort | | | | Smokers | | | |
|  | | Number | Estimates using the extrapolation based on the first 8 years of follow-up | 16-year follow-up Kaplan-Meier estimate | Relative bias | Number | Estimates using the extrapolation based on the first 5 years of follow-up | 8-year follow-up Kaplan-Meier estimate | Relative bias |
|  |  |  | *mean (SE) months* | *mean (SE) month* | *%* |  | *mean (SE) months* | *mean (SE) month* | *%* |
| SCLC | Limited | 1094 | 19.4 (1.0) | 20.5 (1.1) | -5.8 | 710 | 23.7 (1.3) | 23.9 (1.0) | -0.9 |
|  | Extensive | 2402 | 8.4 (0.3) | 8.9 (0.3) | -4.9 | 1772 | 8.9 (0.3) | 9.4 (0.3) | -5.3 |
| SqCC | I | 990 | 73.1 (2.5) | 73.9 (2.2) | -1.1 | 623 | 56.1 (1.6) | 57.2 (1.5) | -1.9 |
|  | II | 486 | 51.5 (3.2) | 53.7 (2.9) | -4.1 | 495 | 42.9 (1.6) | 44.6 (1.7) | -3.9 |
|  | IIIA | 899 | 26.1 (1.2) | 27.1 (1.4) | -3.7 | 775 | 26.4 (1.2) | 26.9 (1.1) | -1.9 |
|  | IIIB | 1965 | 17.9 (0.8) | 18.9 (0.8) | -5.5 | 912 | 17.7 (0.8) | 18.4 (0.7) | -3.5 |
|  | IV | 3104 | 10.9 (0.4) | 11.5 (0.4) | -5.2 | 1918 | 9.6 (0.3) | 10.3 (0.3) | -6.8 |
| Adenocarcinoma | I | 2347 | 104.4 (1.7) | 109.6 (1.6) | -4.8 | 1147 | 70.6 (1.3) | 72.2 (1.0) | -2.3 |
|  | II | 499 | 66.6 (2.7) | 67.8 (2.7) | -1.7 | 302 | 52.8 (2.0) | 54.2 (2.1) | -2.6 |
|  | IIIA | 1059 | 44.4 (1.4) | 46.8 (1.6) | -4.9 | 481 | 41.3 (1.6) | 43.5 (1.6) | -5.1 |
|  | IIIB | 3004 | 25.2 (0.6) | 26.5 (0.7) | -5.1 | 534 | 26.4 (1.3) | 26.8 (1.2) | -1.5 |
|  | IV | 10,668 | 18.0 (0.2) | 19.0 (0.3) | -5.3 | 4836 | 15.7 (0.3) | 16.4 (0.3) | -4.2 |
| Other non-SqCC | I | 423 | 69.0 (3.3) | 73.6 (3.5) | -6.3 | 165 | 59.0 (3.2) | 60.0 (2.9) | -1.6 |
|  | II | 167 | 43.2 (4.0) | 40.6 (4.0) | 6.4 | 104 | 28.0 (3.8) | 29.5 (3.2) | -5.0 |
|  | IIIA | 414 | 26.2 (2.2) | 26.7 (2.0) | -1.9 | 156 | 28.9 (2.6) | 29.4 (2.5) | -1.6 |
|  | IIIB | 1114 | 15.9 (0.9) | 17.1 (0.9) | -7.1 | 219 | 16.2 (1.6) | 16.1 (1.5) | 1.0 |
|  | IV | 3792 | 11.1 (0.4) | 11.8 (0.4) | -5.6 | 1066 | 8.1 (0.5) | 8.6 (0.5) | -5.7 |
| SCLC—small-cell lung cancer; SqCC—squamous-cell non-small-cell lung cancer. | | | | | | | | | |
